# Elevated Human Telomerase Reverse Transcriptase Gene Expression in Blood Cells Associated with Chronic Arsenic Exposure in Inner Mongolia, China

**DOI:** 10.1289/ehp.11532

**Published:** 2008-10-02

**Authors:** Jinyao Mo, Yajuan Xia, Zhixiong Ning, Timothy J. Wade, Judy L. Mumford

**Affiliations:** 1 Center for Environmental Medicine, Asthma and Lung Biology, University of North Carolina-Chapel Hill, Chapel Hill, North Carolina, USA; 2 Inner Mongolia Center for Endemic Disease Control and Research, Huhhot, Inner Mongolia, China; 3 Ba Men Anti-epidemic Station, Lin He, Inner Mongolia, China; 4 National Health and Environmental Effects Research Laboratory, U.S. Environmental Protection Agency, Research Triangle Park, North Carolina, USA

**Keywords:** arsenic, blood, drinking water, gene expression, hTERT, humans, mRNA, nail, telomerase, skin hyperkeratosis

## Abstract

**Background:**

Arsenic exposure is associated with human cancer. Telomerase-containing human telomerase reverse transcriptase (hTERT) can extend telomeres of chromosomes, delay senescence, and promote cell proliferation leading to tumorigenesis.

**Objective:**

The goal of this study was to investigate the effects of As on hTERT mRNA expression in humans and *in vitro*.

**Method:**

A total of 324 Inner Mongolia residents who have been exposed to As via drinking water participated in this study. Water and toenail samples were collected and analyzed for As. Blood samples were quantified for hTERT mRNA expression using real-time polymerase chain reaction. The hTERT mRNA levels were linked to water and nail As concentrations and skin hyperkeratosis. Human epidermal keratinocytes were treated with arsenite to assess effects on cell viability and *hTERT* expression *in vitro*.

**Results:**

hTERT mRNA expression levels were significantly associated with As concentrations of water (*p* < 0.0001) and nails (*p* = 0.002) and also associated with severity of skin hyperkeratosis (*p* < 0.05), adjusting for age, sex, smoking, and pesticide use. Females showed a higher slope than males (females: 0.126, *p* = 0.0005; males: 0.079, *p* = 0.017). In addition to water and nail As concentrations, age (*p* < 0.0001) and pesticide use (*p* = 0.025) also showed significant associations with *hTERT* expression. The *hTERT* expression levels decreased with age. Tobacco smoking did not affect *hTERT* expression (*p* = 0.13). *hTERT* expression was significantly correlated with *OGG1* and *ERCC1* expression. The *in vitro* results also showed a dose–response relationship between arsenite concentrations and *hTERT* expression and reached the peak at 1 μM.

**Conclusions:**

*hTERT* expression was associated with As exposure *in vivo* and *in vitro*. The increased *hTERT* expression may be a cellular response to genomic insults by As and may also indicate that As may function as a tumor promoter in carcinogenesis in humans.

Chronic arsenic exposure via drinking water is an environmental health concern worldwide. Epidemiologic studies have shown that exposure to As is associated with elevated risk of skin, lung, and bladder cancers and other chronic health effects, including cardiovascular and peripheral vascular disorders, peripheral neuropathy, and diabetes (Abernathy et al. 2003; Yoshida et al. 2004). Proposed modes of action for As effects include perturbations in signal transduction pathways, DNA methylation, DNA repair, DNA damage, chromosome abnormalities, oxidative stress, and co-carcinogenicity (Hughes 2002; Schoen et al. 2004). The molecular events from chronic exposure to As in humans, especially at low doses, are not well understood.

Telomeres, located at the ends of chromosomes, play an important role in maintaining genomic stability (Shay et al. 2001). Deregulation of telomere length has been implicated in human cancer and aging (Djojosubroto et al. 2003). Telomere maintenance is closely regulated by telomerase and other telomere-associated proteins. Human telomeres consist of repeated units of the hexanucleotide TTAGGG (Blackburn 2000a). Under normal conditions, because of the inability of DNA synthesis to copy the 3′-termini of chromosomes during cell replication, there is a gradual loss of telomeric sequence. The telomeres shorten with progressive cell division, and eventually senescence or apoptosis takes place. Telomerase is capable of adding telomeric sequences (TTAGGG repeats) to the ends of chromosomes, thereby preventing the loss of chromosomes at each cell division. Human telomerase consists of an RNA subunit [human telomerase RNA (hTR)] and the protein components, including an enzymatic subunit (telomerase reverse transcriptase or hTERT) and dyskerin (Cohen et al. 2007). Most normal somatic cells contain minimal or no detectable telomerase activity, whereas immortal and tumor cells exhibit significant levels of telomerase activity and show no net loss of telomere length during proliferation. The evaluation of telomerase in human cancer has been proposed for diagnostic and therapeutic purposes. hTERT is associated with telomerase activity and is expressed in about 85% of human cancers but not in most normal somatic cells (Ito et al. 1998; Takakura et al. 1998). Peripheral blood cells express low levels of telomerase activity, and the expression of the human telomerase reverse transcriptase gene [*hTERT*; GenBank accession no. AF015950; National Center for Biotechnology Information (NCBI) 2009] can be elevated upon activation of cells (Broccoli et al. 1995; Counter et al. 1995). Thus, peripheral blood cells may be useful as a surrogate tissue for evaluating the effects of As in human cells *in vivo*.

Arsenic naturally occurs in groundwater in Bayingnormen (Ba Men), a region located in western Inner Mongolia, China (Guo et al. 2003). The Ba Men residents, a stable population consisting mostly of farmers, have been exposed to a wide range of As levels [below the limit of detection (LOD) to 1.8 mg/L] mainly via drinking the water from artesian wells for > 20 years (Ma et al. 1999). In Ba Men, > 300,000 people have been chronically exposed to As, especially in three counties of Hangjin Hou, Lin He, and Wu Yuan. Health effects at multiple end points, including dermal, neurologic, cardiovascular, and peripheral vascular effects, have been reported. More than 80% of the families own individual wells, so it is possible to assess As exposure at the individual level. In China, Ba Men is well known for its abundance in agriculture products both for commercial use and for personal consumption. Seafood consumption is not common, and As-containing pesticides have not been used in Ba Men (Mo et al. 2006). This population provides a good opportunity to investigate health effects of chronic As exposure via drinking water and to evaluate the application of biomarkers for assessing As exposure and health effects. Previously, we showed that As exposure in this population is associated with increase in mRNA levels of an oxidative-response DNA repair gene, *OGG1* (8-oxoguanine DNA glycosylase gene; GenBank accession no. AB000410.1), and a nucleotide excision repair gene, *ERCC1* (excision repair cross-complementing rodent repair deficiency, complementation group 1; GenBank accession no. BC008930) (Mo et al. 2006; Mo J, unpublished data). These results indicate that chronic As exposure induces DNA repair responses. Studies have shown the association of *hTERT* with DNA repair (Chan and Blackburn 2002; Masutomi et al. 2005). Increase in *hTERT* expression accelerates the repair of DNA double-strand breaks induced by ionizing radiation and also the repair of oxidative DNA adducts due to cisplatin exposure (Sharma et al. 2003). Suppression of the *hTERT* expression in human fibroblasts decreases the cellular response to DNA double-strand breaks, exhibits increased radio-sensitivity, and greatly reduces DNA repair. Thus, alteration in *hTERT* expression may be associated with cellular response to genomic insults in human cells.

Previously, we reported that As at low concentrations (≤ 1 μM) increases telomerase activity/expression and induces cell proliferation in human epidermal keratinocytes and leukemia cells *in vitro*, whereas at higher concentrations (> 1 to 40 μM) As decreases telomerase activity/expression and induces cell apoptosis (Zhang et al. 2003). Because telomeres, which are regulated by telomerase activity, are critical in maintaining the integrity of chromosomes, and because As is known for its clastogenicity, we conducted this study to investigate As effects on *hTERT* expression *in vitro* in human epidermal keratinocytes and also *in vivo* in the Inner Mongolian population. Skin hyperkeratosis is an important characteristic of chronic As exposure in Inner Mongolia (Ma et al. 1999). In this study, we also linked *hTERT* expression to skin hyperkeratosis.

## Materials and Methods

### Study subjects

A total of 324 study subjects from the subvillages of *a*) Wulan, Jianshe, Fengchan, and Xinyao located in Sha Hai Village, Hangjin Hou County, and *b*) Miaohao and Xigelian located in Sheng Feng Village, Wu Yuan County, in Ba Men, Inner Mongolia, were recruited as described previously (Mo et al. 2006). Before subject selection, well water samples from the homes of residents of these subvillages were collected and analyzed for As concentrations. The study subjects were selected according to the criteria set for study design focusing on the effects of low doses of As (≤ 200 μg/L) on *hTERT* expression. The criteria included *a*) approximately 70% of subjects with As exposure levels from nondetectable to 200 μg/L, 30% with As exposure levels > 200 μg/L, and As exposure for at least 5 years; *b*) an approximately equal number of males and females and the ratio of smokers and nonsmokers about 1:2; and *c*) age ranging from 11 to 65 years. Questionnaires were administered to all participants to obtain demographic information, history of well use, diet, smoking, occupation, pesticide use, and medical information. Because only a limited number of subjects (nine subjects) were ex-smokers, we defined smokers as those who reported current smoking and we included ex-smokers with the nonsmokers. Study subjects were asked if they had used pesticides in the past 5 years (yes/no). The pesticides commonly used in this study site are organophosphate, organochlorine, and nitrogen-containing compounds. Skin hyperkeratosis was determined as the presence of benign wartlike growths in skin, diagnosed based on China’s national standards for diagnosis of arsenicosis (People’s Republic of China 2000). To protect human subjects, this study was conducted according to the recommendations of the Declaration of Helsinki (World Medical Association 1989) for international health research. All subjects gave written informed consent to participate in this study. This research protocol met the requirements for protection of human subject certification and was approved by the U.S. Environmental Protection Agency.

The Ba Men residents use well water for drinking almost all the time, with the exception of rare occasions when they are away from home. Commercial bottled water is very seldom used. Arsenic levels in well water may vary in the dry (winter) and rainy (spring/summer) seasons. The As exposure levels among the study subjects are constant most of the time, especially within the same season. For this reason, in this study water, blood, and toenail samples were collected from each individual within 1–2 days. All samples were collected from all subjects within 1 month in the winter.

### Water collection and analysis

A sample of drinking water (from the well) of each subject was collected as described previously (Mo et al. 2006). Briefly, the water samples were collected in acid-washed tubes and analyzed for total As using inductively coupled plasma mass spectrometry (ICPMS) (Gong et al. 2006). For quality assurance, we periodically analyzed a standard reference material (SRM 1643d water; National Institute of Standards and Technology, Gaithersburg, MD, USA). The result from 44 analyses of SRM 1643d (certified value, 56.0 μg/L) was 57.7 ± 2.6 μg/L. We also periodically analyzed water samples spiked with As and method blanks. The LOD for As in water by ICPMS in this study was 0.1 μg/L.

### Toenail collection and analysis

A toenail sample from each study subject was collected as described previously (Mo et al. 2006). The nail samples were collected and stored in zip-type plastic bags and shipped via air to the United States. The nail samples were cleaned by sonication in HPLC-grade water to remove the water-soluble contaminants. After removing the water, acetone was then added to remove organic contaminants from the nail surface (Schmitt et al. 2005). Nail samples were analyzed for As content by instrumental neutron activation analysis (INAA) at the Nuclear Services Department of North Carolina State University (Raleigh, NC, USA) (Heydorn 1984). The certified standard reference materials (DORM2, DOLT3; National Research Council Canada, Ottawa, Ontario, Canada) and method blanks were run with the human nail samples. The values measured for the reference material controls were within the 15% variance of the standards. The LOD for determining As in nails by INAA was 0.012 μg/g.

### Blood collection and RNA isolation

We used peripheral blood cells as a surrogate for target cells in this study. Sample collection and analysis methods have been previously described (Mo et al. 2006). Briefly, a 2.5 mL peripheral blood sample was collected into a PAXgene blood RNA tube containing the RNA-stabilizing agent (Qiagen, Valencia, CA, USA). The blood samples were transported to the United States by air on ice packs and stored at −80°C until RNA isolation. Total RNA was isolated from whole blood using PAXgene blood RNA kits according to the manufacturer’s instructions (Qiagen). We checked the integrity of RNA by RNA electrophoresis in agarose gels and by using an Agilent 2100 Bioanalyzer (Agilent Technologies, Santa Clara, CA, USA). We found no degradation of RNA as indicated by intact 18S and 28S RNA in RNA electrophoresis and by bioanalyzer.

### Quantitative real-time polymerase chain reaction (PCR)

The first-stranded cDNA synthesis was conducted in a reaction mixture with total volume 50 μL containing 1X buffer, 2.5 μM random primers, 5 mM dithiothreitol, 500 μM deoxyribonucleotide triphosphate, 40 U RNase inhibitor, 200 U SuperScript III reverse transcriptase (Invitrogen, Carlsbad, CA, USA), and 500 ng total RNA. The reactions were performed at 25°C for 10 min, 42°C for 60 min, and then heated to 85°C for 5 sec to inactivate the reverse transcriptase. We used a total of 4 μL cDNA for each real-time PCR measurement in a total volume of 50 μL. We performed real-time PCR with an ABI Prism 7700 Sequence Detection System (Applied Biosystems, Foster City, CA, USA) to quantitatively compare the mRNA levels as described previously (Mo et al. 2006). Probes and primers were obtained from Applied Biosystems (assay IDs: *hTERT*, HS00162669_m1; β*-*actin, 4326315E). Real time PCR was performed in duplicate with 2X TaqMan Universal PCR Master Mix (Applied Biosystems). The thermal cycling conditions consisted of one cycle each for 2 min at 50°C and 10 min at 95°C and 40 cycles for 15 sec at 95°C and 1 min at 60°C. The hTERT mRNA levels were determined by a relative standard curve method using HT1080 cell RNA as standard (Applied Biosystems 2001). The hTERT mRNA levels were normalized to β*-*actin mRNA levels, which were determined in the same tube as the target gene (*hTERT*). For quality control, distilled water and blood total RNA were used as negative controls. HT1080 cDNA and cDNA from a human blood sample were used as positive controls in each assay. The SD on repeated measurements of HT1080 cDNA and the blood cDNA in the repeated experiments was < 10%. We conducted repeated measurements in 10% of the total blood samples. Samples showing threshold cycle (C_T_) ≥ 36 were considered to be < LOD.

### In vitro *studies.*

The human epidermal keratinocyte cell line (HaCaT) used in this study was a gift from N.E. Fusenig (German Cancer Institute, Heidelberg, Germany). We cultured cells in Dulbecco’s modified Eagle’s medium supplemented with 10% fetal bovine serum (Invitrogen), penicillin (100 U/mL), and streptomycin (100 μg/mL) and maintained them at 37°C in 95% air and 5% CO_2_. We conducted the MTT [3-(4,5-dimethyl-thiazol-2-yl)-2,5-diphenyltetrazolium bromide] assay using cell proliferation assay kits (Roche, Indianapolis, IN, USA) to assess As effects on cell viability (or cytotoxicity), following the manufacturer’s instructions. Briefly, 1 × 10^4^ HaCaT cells were seeded in 96-well plates in 0.2 mL media. After 24 hr, we added sodium arsenite (Sigma Chemical Co., St. Louis, MO, USA) to the cultures to reach the final concentrations of 0.1–10 μM and incubated them for 3 days. Cell viability was measured at 560 nm and expressed as the percentage of controls. We conducted the assay in triplicate wells for each treatment and conducted a total of three experiments for the assay.

For the hTERT mRNA assay, after seeding the cells for 24 hr, we treated them with sodium arsenite for 72 hr at concentrations 0–10 μM. Total RNA was isolated from cultures of control and arsenite-treated cells using the SV Total RNA Isolation System (Promega, Madison, WI, USA). RNA quality was assessed by analysis with an Agilent 2100 Bioanalyzer. The assay for hTERT mRNA expression of the *in vitro* samples was conducted in the same manner as for the human blood samples described above. We included three replicates for each dose for each experiment, conducting a total of three experiments (nine replicates total for each dose) for this assay.

### Statistical analysis

We transformed data by taking the log (base_10_) for data analysis to reduce the skewness evident in the *hTERT*, *ERCC1*, and *OGG1* expression and water and nail As measures. Mean *hTERT* expression was compared in subgroups using *t*-tests or *F*-tests from analysis of variance models. We used linear regression models and Spearman correlation coefficients to evaluate the association between continuous dependent variables (*hTERT* expression) and independent predictor variables. Interaction terms between key covariates and predictors were included in regression models. We constructed multivariate models, controlling for variables related to the outcome or predictor variable. For analysis of *in vitro* data, Shapiro-Wilk tests for normality were conducted to confirm the approximate normal distribution of the replicates. Once approximate normality was confirmed, we used *t*-tests with unequal variance to quantify differences in *hTERT* expression compared with the baseline group across different levels of As concentrations. We used multivariate regression models to compare *hTERT* expression (log_10_ transformed) among those with skin hyperkeratosis, controlling for age, sex, smoking, and pesticide use. Skin hyperkeratosis was classified as “absent,” “mild,” “moderate,” or “severe.” In these models, we used *hTERT* expression as the dependent variable, and we used indicator variables to represent the degree of skin hyperkeratosis. Group differences in *hTERT* expression were assessed based on the significance of the regression coefficients. Statistical analyses were conducted using SAS, version 9.1 (SAS Institute Inc., Cary, NC, USA) and Stata version 10.1 (StataCorp, College Station, TX, USA).

## Results

### Study subjects

Demographic information, tobacco smoking, water As concentrations, pesticide use, and skin lesion occurrence for the study subjects are shown in Table 1. Of the 324 study subjects (163 males and 161 females), 79% were farmers. Among them, 218 (67%) were nonsmokers and 106 (33%) were smokers. The age of the subjects ranged from 11 to 61 years, with a mean ± SD of 36 ± 14 years. Most of the subjects (76%) were exposed to water As concentrations of 0.34–200 μg/L; 12% of subjects were exposed to 201–300 μg/L, and 12% to 301–826 μg/L. Nail As concentrations ranged from 0.24 to 63.14 μg/g. All water and nail As concentrations were > LOD. The mean (± SD) length of As exposure was 13 ± 6 years. Forty-eight percent of the subjects reported having used pesticides in the past 5 years.

### hTERT *expression and As exposure.*

**Figure 1:**
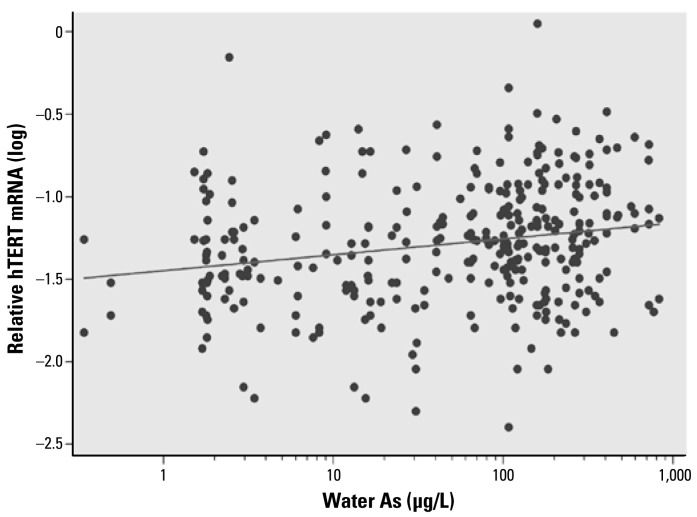
Association between hTERT mRNA levels and water As concentrations. Adjusting for age, sex, smoking, and pesticide use, hTERT mRNA levels were significantly associated with water As concentrations using a linear regression model: *n* = 323, *p* < 0.0001*,* hTERT*(*log) = −1.15 + 0.102 × water As(log); Spearman *r* = 0.2313, *p* < 0.0001.

**Figure 2:**
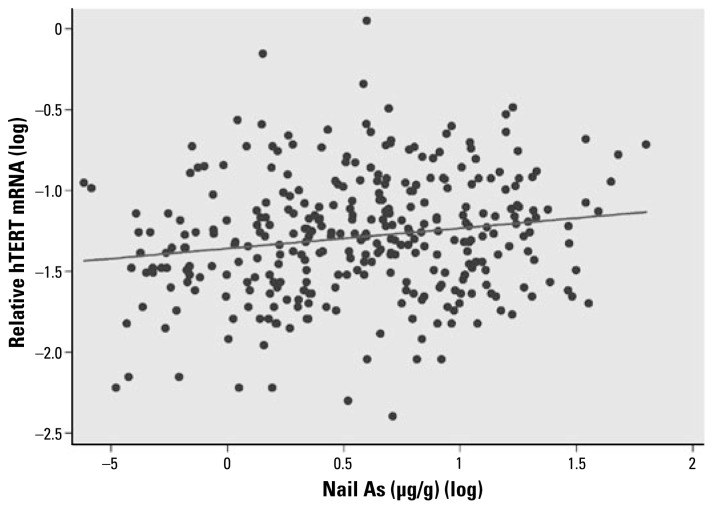
Association between hTERT mRNA levels and nail As concentrations. Adjusting for age, sex, smoking, and pesticide use, hTERT mRNA levels were significantly associated with nail As concentrations: *n* = 322, *p* = 0.002, hTERT*(l*og) = −1.06 + 0.120 × nail As(log); Spearman *r* = 0.1674, *p*= 0.0025.

**Figure 3:**
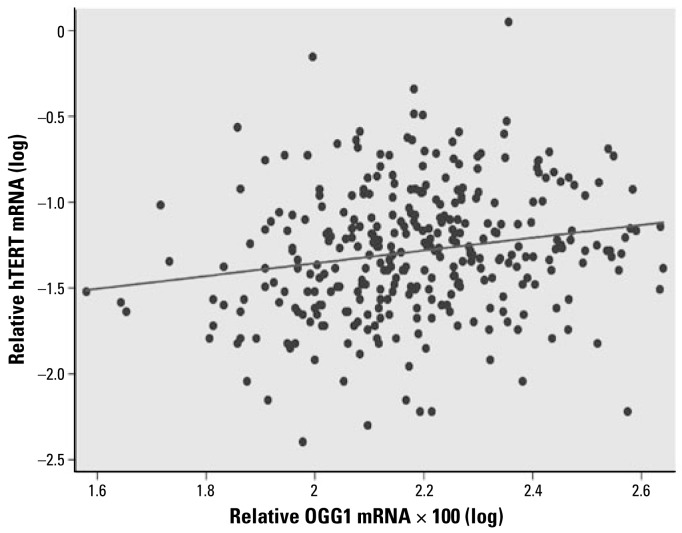
Correlation between hTERT and OGG1 mRNA levels in human blood cells (*n* = 323, Spearman *r* = 0.212, *p* = 0.0001). Based on linear predictions adjusted for age, sex, smoking, and pesticide use, hTERT(log) = −1.85 + 0.39 × OGG1 ×100(log).

**Figure 4:**
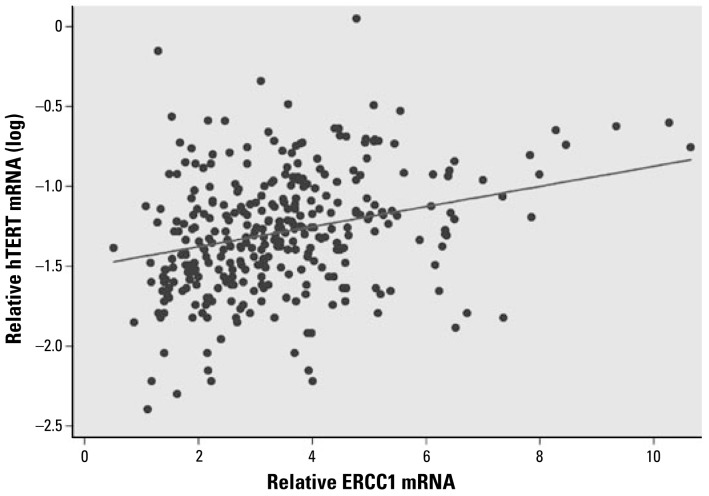
Correlation between hTERT and ERCC1 mRNA levels in human blood cells (*n* = 323, Spearman *r* = 0.329, *p* < 0.0001). Based on linear predictions adjusted for age, sex, smoking, and pesticide use, hTERT(log) = −1.27 + 0.0624 × ERCC1.

All the blood samples showed detectable levels of hTERT mRNA by real-time PCR. The mRNA levels of *hTERT* in the blood cells from the study subjects were highly associated with the concentrations of water As (slope = 0.102, *p* < 0.0001), adjusting for age, sex, smoking, and pesticide use (Figure 1). When we analyzed the data separately by sex, both males and females showed significant associations between *hTERT* expression and water As concentrations (slope: males, 0.079, *p* = 0.017; females, 0.126, *p* = 0.0005). Females showed a higher slope than males in hTERT mRNA levels. The relative mean levels of hTERT mRNA were 0.073 for males and 0.079 for females. In our previous study, we found a strong positive correlation between toenail and drinking-water As concentrations (Spearman *r* = 0.882, *p* < 0.0001) in these subjects (Mo et al. 2006). In the present study, *hTERT* expression was also significantly associated with toenail As concentrations (slope = 0.120, *p* = 0.002; Figure 2). In addition to As exposure, age (slope = −0.0094, *p* < 0.0001) was also significantly associated with *hTERT* expression levels. Among the three age groups, the mean levels of hTERT were lowest in the > 50-year age group (0.0572), followed by the 19- to 49-year age group (0.0700) and then the 11- to 18-year age group (0.1154). Pesticide use also showed significant associations with *hTERT* expression (*p* = 0.025). Tobacco smoking was not significantly associated with hTERT mRNA expression (*p* = 0.13). The interaction tests showed no differences in the slope of hTERT levels and water As concentrations by sex, age, smoking, or pesticide use (*p* = 0.268, 0.838, 0.248, and 0.632, respectively). Spearman correlation analysis showed that hTERT mRNA levels in blood cells were positively correlated with both OGG1 and ERCC1 mRNA levels (Spearman *r* = 0.212, *p* = 0.0001 for *hTERT* and *OGG1* and *r* = 0.329, *p* < 0.0001 for *hTERT* and *ERCC1*; Figures 3 and 4).

### hTERT *expression in keratinocytes* in vitro

**Figure 5:**
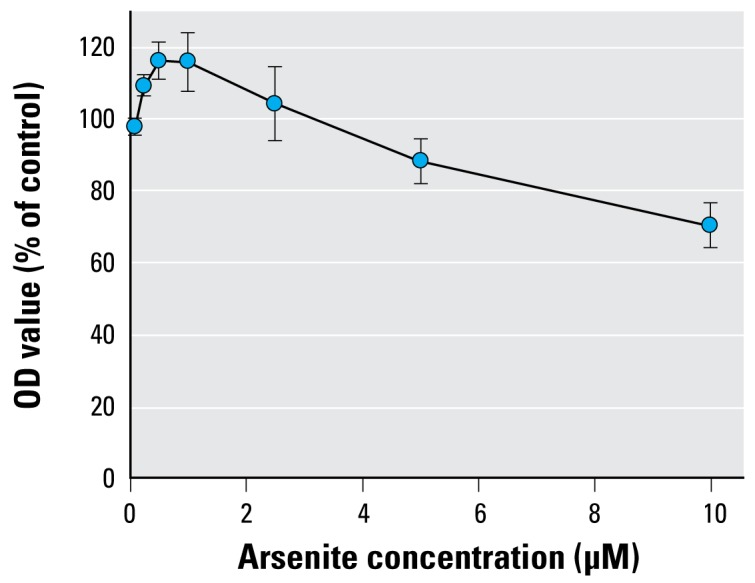
Arsenite effects on cell viability by MTT assay. Cell viability was determined by optical density (OD) at 560 nm and expressed as percentage of controls. Data shown are mean ± SD of a total of nine replicates from three experiments.

**Figure 6:**
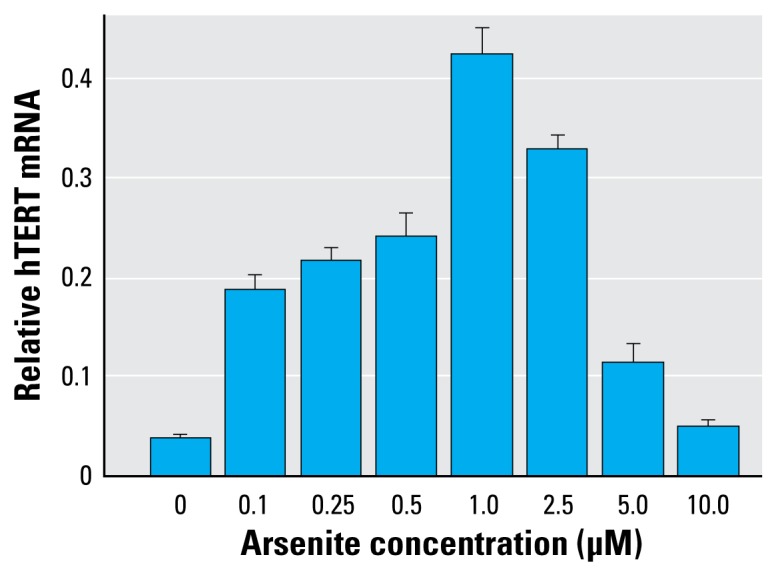
hTERT mRNA levels in HaCaT cells exposed to arsenite *in vitro*. Each bar represents the mean ± SD of three experiments (nine replicates total for each dose). All of the treated cells were significantly different from the controls (*p* < 0.001). hTERT mRNA levels showed a positive dose response up to 1 μM (trend analysis, *p* < 0.0001) and then decreased as the dose increased (trend analysis, *p* < 0.0001).

Results from the MTT assay showed that cell growth of HaCaT cells increased with As treatment at low concentrations, with maximal effects between 0.5 and 1.0 μM, and then decreased as arsenite concentrations increased (Figure 5). For hTERT mRNA levels, all the As-treated cells were significantly different from the control cells (*p* < 0.001; Figure 6). The mRNA levels of *hTERT* increased as arsenite concentrations increased in a dose–response relationship up to 1.0 μM and then decreased as the arsenite concentration increased. The *p*-value for the positive trend from 0 to 1.0 μM was significant (slope = 0.079, *p* < 0.0001), and then the levels from 2.5 to 10 μM showed significant downtrend (slope = −0.091, *p* < 0.0001).

### hTERT *expression and skin hyperkeratosis.*

Among the 322 study subjects with available skin hyperkeratosis information, 224 subjects showed absence of skin hyperkeratosis, 68 showed mild hyperkeratosis, 24 showed moderate hyperkeratosis, and 6 showed severe hyperkeratosis. After controlling for age, sex, smoking, and pesticide use, those with moderate to severe skin hyperkeratosis had higher mean levels of *hTERT* expression compared with those with either no skin lesions (*p* = 0.047) or with mild skin hyperkeratosis (*p* = 0.022). Those with mild skin hyperkeratosis had *hTERT* expression levels similar to those without skin hyperkeratosis (*p* = 0.432).

## Discussion

Because activation of telomerase may play a role in DNA repair and is necessary for sustaining tumor growth and promoting tumorigenesis, in the present study we examined the effects of As on hTERT mRNA expression in humans *in vivo* and *in vitro*. In the *in vivo* human study, we examined the effects of As exposure on hTERT mRNA levels in blood cells from individuals exposed to a wide range of As levels via drinking water in Inner Mongolia. All the blood samples showed detectable levels of hTERT mRNA by real-time PCR. The *hTERT* expression in blood cells was highly associated with the concentrations of As in drinking water. Toenail As concentration has been reported to reflect chronic exposure of As up to 6–12 months (Slotnick and Nriagu 2006). In the present study *hTERT* expression was also associated with the concentrations of As in toenails. We substantiated the human *in vivo* results with the *in vitro* studies, which showed induction of *hTERT* expression by As in human keratinocytes. hTERT mRNA expression was also associated with age but not smoking. We found that *hTERT* expression decreased with age, which is in agreement with previous reports showing that telomeres progressively shorten in most human cells with increased age and are related to lacking active telomerase in cells (Blackburn 2000b). We also found an association between *hTERT* levels and the degree of skin hyperkeratosis.

When human cells are exposed to environmental contaminants, adaptive responses to stress may occur involving adjustment in the levels or activity of the genome-protecting protein machinery such as DNA repair, cell cycle checkpoints, and apoptosis (Hartwig et al. 2002; Sen et al. 2007). These are the survival mechanisms for maintaining healthy cellular status. Cadmium has been shown to induce telomerase activity by evoking a stress response (Alfonso-De Matte et al. 2002a). Harrington and Greider (1991) showed that telomerase may play some role in DNA repair and chromosome healing. Increased telomerase activity has been found in sun-exposed skin and cancer cells that received X-irradiation (Taylor et al. 1996; Ueda et al. 1997). The cells lacking *hTERT* exhibit increased radiosensitivity, diminished capacity for DNA repair, and fragmented chromosomes, suggesting that *hTERT* may play some role in DNA repair and in maintaining chromosome integrity. Previously, we observed that As exposure is associated with elevation of mRNA levels in two DNA repair genes, *OGG1* and *ERCC1*, in blood cells in this population (Mo et al. 2006; Mo J, unpublished data). In the present study, we found that *hTERT* expression was positively correlated with expression of these two genes. It has been reported that ERCC1/XPF nucleotide excision repair endonuclease, which binds with TRF2 (a telomere-binding protein), plays an important role in maintaining telomere integrity (Zhu et al. 2003; Wu et al. 2007). The increased *ERCC1* expression may reflect the DNA repair response to As exposure by forming complexes with TRF2 to protect telomeres in human cells. Thus, the increase in *hTERT* expression may function as a repair response to DNA or chromosome damage from oxidative stress due to As exposure.

The increase in *hTERT* expression may also suggest that telomerase may function as a tumor promoter in humans. The adaptive responses mentioned above can also paradoxically generate genotoxic stress and promote tumorigenesis (Hofseth 2004). Telomere maintenance by telomerase is the primary mechanism by which cancer cells overcome mortality and extend their life span. Most human cancers express telomerase, which is critical in maintaining telomere length and thus in promoting cell proliferation leading to tumor growth. Our previous *in vitro* studies showed that, depending on the concentrations of the treatments, arsenite altered telomere length and telomerase activity and had effects on cell proliferation and apoptosis in human keratinocytes and leukemia cells. At ≤ 1 μM, arsenite induced *hTERT* activity and protein expression and promoted cell growth (Zhang et al. 2003). Chou et al. (2001) found that telomerase activity and hTERT mRNA expression were inhibited at 0.75 μM and 2 μM in NB4 cells treated with arsenite. In the present study, *hTERT* expression increased with As exposure both *in vivo* and *in vitro*. The discrepancy could be due to the different responses to As cytotoxicity by different cell lines.

The molecular mechanisms of increasing *hTERT* gene expression by As as shown in this study are unclear. Multiple mechanisms may exist to regulate *hTERT* gene transcription, resulting in repression or activation of telomerase activity in cells (Horikawa and Barrett 2003). Studies have shown that As induced a number of gene expression alterations, including DNA repair response, oxidative stress, and signal transduction pathways by direct action or redox control of regulatory molecules (Germolec et al. 1996; Liu et al. 2001). The mitogen-activated protein kinases (MAPKs) regulate the signal transduction pathways in response to a variety of extra cellular stimulants and mediate the regulation of various gene expressions. The c-*Jun* N-terminal kinases (JNKs) and p38 kinase in MAPK pathways can respond to stress stimulation and result in cell proliferation, tumorigenesis, and/or apoptosis. Arsenic has been reported to induce activation of JNKs and p38 kinase in human embryonic kidney cells and mouse epidermal JB6 cells (Huang et al. 1999). Activation of JNKs has also been shown to induce *hTERT* expression in ovarian epithelial cells (Alfonso-De Matte et al. 2002b, 2004). The *hTERT* promoter contains SP1 and c-Myc sites and is essential for *hTERT* gene transcription activation (Kyo et al. 2000). Short-term treatment with As (0.1–5 μM) increases expression and activation of c-*Fos* and c-*Jun* (Hu et al. 2002). In this study, the increased *hTERT* expression in blood cells of the study subjects was likely due to activation of JNKs induced by As exposure and subsequent formation of c-Jun protein complexes that bind to SP1 sites present in the *hTERT* promoter and then up-regulate *hTERT* expression.

The observed elevated expression of *hTERT* shown in the present study could also be regulated by other mechanisms. The *hTERT* gene promoter is a target of hormone carcinogenesis in humans. There could be a link between hormone-induced carcinogenesis and the *hTERT* gene promoter (Boggess et al. 2006). There is a putative estrogen response element in the *hTERT* promoter (Nanni et al. 2002). It is possible that estrogen receptor α can bind to this element in *hTERT* promoter and then activate *hTERT* transcription (Kyo et al. 1999; Misiti et al. 2000). Animal studies have shown that mRNA levels of estrogen receptor α were 3.1-fold higher in liver of As-exposed mice than in those of controls (Waalkes et al. 2004). Our studies in this population showed that As exposure induces estrogen receptor α gene expression (Xia Y, unpublished data). Therefore, the increased hTERT mRNA expression may be linked to As-induced estrogen receptor α expression in humans. In the present study, hTERT mRNA levels in blood cells were also associated with pesticide use. This is in agreement with a report showing that hTERT mRNA expression levels were increased in mouse mammary glands and human mammary gland adenocarcinoma (MCF-7) cells treated with the organochlorine pesticide endosulfan (Je et al. 2005). Because the pesticide is a known estrogen agonist, it is believed that the estrogen-responsive element in *hTERT* gene promoter may be responsible for the transcriptional activation.

The results of the MTT assay showed that cell growth increased as arsenite concentrations increased up to 1.0 μM and then decreased as arsenite concentrations increased from 2.5 μM to 10 μM. The observed reduced hTERT mRNA expression at high levels of arsenite exposure could result from cytotoxicity of arsenite. This finding was consistent with our previous report in HaCaT cells and HL60 cells (Zhang et al. 2003). High levels of arsenite are believed to induce reactive oxygen species, which may play an important role in inhibition of *hTERT* transcription (Ammendola et al. 1994).

Regarding water As speciation in the present study, we used arsenite in the form of As(III) in the *in vitro* study, and our previous speciation study of the water samples from Ba Men showed that the soluble arsenicals in well water contained 65% As(III) and 35% As(V) (Gong et al. 2006). Depending on the storage time of well water, As(V) concentrations may increase. Even though there may be some differences in As speciation of water used in human and *in vitro* studies, As(V) in drinking water after being ingested is reduced to As(III) and then methylated (Kitchin 2001).

In the *in vitro* study, we observed the downward trend of *hTERT* expression levels starting at 2.5 μM, which may be due to cytotoxicity induced by As. We did not find this downward trend in the human blood study. This may be due to the low levels of As concentrations in human blood. In this study, we did not determine As concentrations in blood. However, the blood As levels have been reported to be much lower than As concentrations in drinking water. Wu et al. (2001) reported that the total concentrations of As in well water of Taiwan ranged from ≤ 10 μg/L to > 300 μg/L and blood As levels ranged from 7.2 to 11.4 μg/L (0.1–0.15 μM). In Bangladesh, the As well-water levels ranged from 20 to > 2,000 μg/L and total blood As levels ranged from 0.05 to 1.2 μM (Snow et al. 2005). If these blood levels are applicable to the Inner Mongolia population exposed to As, the blood concentrations in our study subjects may not reach the levels causing cytotoxicity. This may explain why we did not observe the downward trend of *hTERT* expression in human blood study.

There are limitations in the present study. Alteration in *hTERT* expression may not be specific to As exposure because of consumption of As-contaminated water. Other confounding factors may also influence these associations. In addition, because of the limitations of human target cells (lung, skin, and bladder) available for the study, we used blood samples as a surrogate for the target tissues. Regarding recent As exposure in Ba Men, the Chinese government has made efforts to alleviate the As contamination problems in drinking water in this region by changing the water systems to treated water or other alternative wells.

In summary, we examined As effects on *hTERT in vitro* and *in vivo*. This is the first study showing that hTERT mRNA expression in blood cells is positively associated with As exposure in humans. *hTERT* gene expression also increased with As concentrations *in vitro*. The *hTERT* expression in human blood cells was associated with the skin hyperkeratosis from chronic As exposure. The correlated expressions of *hTERT*, *OGG1*, and *ERCC1* reflects their functional interactions to protect cells against DNA or chromosome damage from As. The observed hTERT mRNA increase represents a human adaptive response to As insult and suggests As may function as a tumor promoter in human carcinogenesis.

## Figures and Tables

**Table 1:** Distribution of study population characteristics (*n* = 324).

Variable	No. of subjects (%)
Sex	
Male	163 (50.3)
Female	161 (49.7)
Tobacco smoking	
Nonsmoker	218 (67.3)
Smoker	106 (32.7)
Age (years)^a^	
11–18	58 (18.0)
19–49	212 (65.6)
> 50	53 (16.4)
Water As concentration (μg/L)	
0.34–50	130 (40.1)
51–200	116 (35.8)
201–826	78 (24.1)
Used pesticides in past 5 years	
Yes	156 (48.1)
No	168 (51.9)
Skin hyperkeratosis^a^	
None	224 (69.6)
With	98 (30.4)

a Some data are missing for age and skin hyperkeratosis.
